# Commentary on the special issue: Leveraging measurement to refine developmental perspectives on psychopathology

**DOI:** 10.1002/mpr.1996

**Published:** 2023-10-31

**Authors:** Daniel S. Pine

**Affiliations:** ^1^ Section on Development and Affective Neuroscience National Institute of Mental Health Intramural Research Program Bethesda Maryland USA

## 1 INTRODUCTION

This Special Issue of International Journal of Methods in Psychiatric Research highlights developmental perspectives on irritability and related dimensions of psychopathology. The issue's papers (Alam et al., 2023; Hirsch et al., 2023; Kirk et al., 2023; Wakschlag et al., 2023; Wigg et al., 2023; Wiggins et al., 2023a, 2023b) extend a solid foundation of research to target questions on pediatric emotional problems and applications of dimensional methods. As such, the papers are fresh and novel while steeped in an important prior history. Papers demonstrate improvements in measurement during the first 5 years of life (Hirsch et al., 2023; Wakschlag et al., 2023; Wiggins et al., 2023a, 2023b) through adolescence (Alam et al., 2023; Kirk et al., 2023; Wakschlag et al., 2023), important areas in need of advancement.

Infancy and preschool are particularly important years in the life of a child. This reflects, at least partly, research findings suggesting that the seeds of later psychopathology germinate during this developmental period. The Special Issue highlights how some forms of psychopathology first manifest with symptoms of irritability, representing expressions of excessive anger and frustration during blocked goal attainment (Alam et al., 2023; Hirsch et al., 2023; Wiggins, Ureña Rosario, Zhang, et al., 2023). However, irritability can represent normative expressions of emotions. Given the breadth of normative behavior that rapidly changes in the first 5 years, clinicians face difficulty in young children separating extreme but normal variation in irritability from clinically significant symptoms in this domain. The first Special Issue paper (Wigg et al., 2023) reviews themes targeted in the other papers, and this Commentary broadly highlights three related major issues: (i) the nature of developmental risk; (ii) measurement challenges in young children, and (iii) relations between early irritability and later psychopathology.

## 2 NATURE OF DEVELOPMENTAL RISK

Special Issue papers focus heavily on the Multidimensional Assessment Profile Scales, Temper Loss scale (MAPS‐Temper Loss) (Kirk et al., 2023; Wakschlag et al., 2023; Wiggins, Ureña Rosario, MacNeill, et al., 2023). This measure quantifies levels of irritability expressed by children, now extended from age 1 year through adolescence. However, the MAPS‐Temper Loss measure arises from a broader series of assessment tools that quantify other aspects of early life psychopathology. This includes low concern for others and aggression. Moreover, other measures in young children quantify aspects of neurodevelopment and learning, based on children's performance on standardized tests. Thus, multiple domains of psychopathology can be quantified in young children, often using unique measurement approaches for each domain.

The contribution from papers in the Special Issue can be more fully appreciated by recognizing the distinct nature of risk associated with specific domains of early‐life psychopathology. The Special Issue focuses on irritability (Alam et al., 2023; Hirsch et al., 2023; Kirk et al., 2023; Wiggins, Ureña Rosario, MacNeill, et al., 2023); in this symptom domain, pathology represents extreme expressions of behavior that many children manifest. Thus, most young children manifest irritability at various times during their lives; irritability in this age group only is pathological when it exceeds these normative expressions. Similar patterns manifest for anxiety, which also is common in young children. Pathological anxiety involves excessive levels of a behavior that can be considered normative in many contexts. The Special Issue reports on an extension of past methods to characterize the dimensional spectra of anxious and depressive behaviors within the developmental context of early childhood (Alam et al., 2023; Kirk et al., 2023; Wakschlag et al., 2023).

As authors of Special Issue papers note in other research, this pattern for irritability and anxiety differs from patterns for other domains. For example, while expressions of irritability and anxiety frequently occur in healthy young children, manifesting low concern for others, another dimension of the MAPS scales, is far rarer in this age group. Almost no child expresses low‐concern behaviors, whereas many children manifest irritability. Thus, the presence of any level of low‐concern behavior can indicate psychopathology, unlike for irritability where only high levels in developmentally unexpected contexts are pathologic. For example, while most young children experience tantrums, less than 10% are reported to have such experiences on a daily basis (Hirsch et al., 2023; Wiggins, Ureña Rosario, MacNeill, et al., 2023). Finally, unlike either irritability or low concern, still other forms of pathology manifests as a failure to exhibit behaviors that typically arise during specific developmental stages. For example, children typically express features of language, motor control, and learning proficiency during relatively precise time windows. When such features manifest outside the typical time windows, clinicians consider the presence of a neurodevelopmental disorder.

Contrasting findings for multiple domains illustrates the varied expressions that psychopathology involves in young children. In some domains, such as irritability as well as many anxious and depressive behaviors, problems present as extreme levels of behaviors that might be considered normative at younger ages. This is where a Special Issue paper demonstrates the added value of age‐specific measures in children of various ages, spanning early‐childhood through adolescence; such benefits are often theorized but rarely empirically validated. In other domains, the presence of some behaviors, even at low levels, can define pathology, whereas the absence of other behaviors can support the same conclusion.

## 3 MEASUREMENT CHALLENGES

Each of the Special Issue papers highlights the measurement challenges confronting scientists who study early life psychopathology. These challenges arise from many factors. This includes the need to utilize multi‐informant assessments, limitations in young children's capacity to describe psychological states, and the rapid pace of development in this age group.

Special Issue papers provide a roadmap for scientists attempting to address measurement challenges in many areas. Thus, papers describe methods from item response theory (IRT) that help scientist tackle some of these challenges (Alam et al., 2023; Kirk et al., 2023; Wakschlag et al., 2023). By dimensionally modeling expressions of underlying latent traits, IRT increases the efficiency of questionnaires to reduce the overall burden placed on patients. Moreover, in other work, the teams contributing papers to the Special Issue have related measures from IRT to behavioral observations of children, exposed to situations in the clinic that evoked expressions of psychopathology. This relates responses on questionnaires to ratings made by health care professionals. Finally, papers in the Special Issue rely on intensive sampling of children's behavior at multiple points in development. This provides age‐specific norms that endow a dynamic perspective on psychopathology. With such intensive sampling, levels of irritability are viewed as pathological relative to age‐specific norms.

The field owes much to the contributions of the Special Issue papers. Few areas of research provide the field with more important tools than studies of measurement properties. Moreover, particularly important tools arise from approaches to measurement that combine sophistication and rigor with creativity to generate new tools. Special Issue papers provide a tour de force concerning the types of approaches that usefully extend measurement to early childhood, and the papers show the fruits of these labors by demonstrating the measures' potential utility.

## 4 EARLY IRRITABILITY AND LATER PSYCHOPATHOLOGY

While many facets of the new Special Issues could satisfy the curious reader, papers by Wiggins and colleagues (Wiggins et al., 2023a, 2023b) as well as by Hirsch and colleagues (Hirsch et al., 2023) provide particularly interesting data. The importance of these papers derives from their use of prospective, longitudinal approaches.

Research throughout the transition between the 20th and 21st Century highlighted the importance of developmental perspectives on psychopathology. Studies during this period followed many children as they passed through adolescence while providing serial assessments of psychopathology. This work established the presence of strong relations among psychopathology expressed during childhood, adolescence, and adulthood. This work tended to emphasize expressions of psychopathology that first manifested during the school age years. This often reflected the availability of measures with acceptable psychometrics in this age group, which were not generally available at earlier ages. With the publication of the Special Issue, more evidence is added to an accumulating series of findings that extends this prior work both earlier (to age 1) and later (through age 17). Much like earlier work in older youth, Special Issue papers demonstrate acceptable psychometrics across these developmental periods (Kirk et al., 2023). Longitudinal data in the Special Issue demonstrate predictive utility from preschool to pre‐adolescent clinical outcomes, with increased odds of subsequent psychopathology (Hirsch et al., 2023; Wiggins, Ureña Rosario, MacNeill, et al., 2023). Such preschool‐age vulnerability indicators, assessed with the new tools, provide important developmental insights.

Findings on longitudinal outcomes in the Special Issue highlight the importance of early childhood irritability. Not only does this domain of psychopathology show continuity with similar forms of psychopathology during school age, but irritability relates to a broad array of problems. This includes symptoms of multiple internalizing disorders, involving expressions of anxiety or mood problems, as well as externalizing disorders, involving expressions of behavioral and neurodevelopmental difficulties. Such broad expressions of risk highlight the clinical importance of early irritability and dimensional approaches in young children and beyond. Not only is this domain important for the impact it has on young children's functioning, but it also can portend multiple areas of lasting difficulty.

## 5 CONCLUSIONS

Journal readers owe considerable gratitude to the authors of the Special Issue papers. These manuscripts draw attention to an important domain of psychopathology, irritability. Moreover, the papers highlight the measurement challenges raised by attempts to study the domain, and they illustrate novel techniques for meeting these challenges, such as IRT measurement techniques. Considerable other work suggests that these approaches could be useful in many areas of psychopathology. Finally, the Special Issue papers extend a focus on development that characterizes considerable research around mental health. Clearly, the importance of school age and adolescence has been recognized for decades in studies of many mental conditions. Moreover, researchers examining facets of neurodevelopmental disorders have long recognized the importance of studying young children. The Special Issue extends this focus applied to the neurodevelopmental disorders to a range of conditions, particularly those related to expressions of irritability with origins in the first 5 years of life.

## CONFLICT OF INTEREST STATEMENT

The authors declare that there are no conflict of interests.

## References

[mpr1996-bib-0001] Alam, T. , Kirk, N. , Hirsch, E. , Briggs‐Gowan, M. , Wakschlag, L. S. , Roy, A. K.*, & Wiggins, J. L.* (2023). Characterizing the spectrum of irritability in preadolescence: Dimensional and pragmatic applications. International Journal of Methods in Psychiatric Research. e1988. 10.1002/mpr.1988 37800620 PMC10654812

[mpr1996-bib-0002] Hirsch, E. , Alam, T. , Kirk, N. , Bevans, K. , Briggs‐Gowan, M. , Wakschlag, L. S. , Wiggins, J. L.*, & Roy, A. K.* (2023). Developmentally specified characterization of the irritability spectrum at early school age: Implications for pragmatic mental health screening. International Journal of Methods in Psychiatric Research. e1985. 10.1002/mpr.1985 37712753 PMC10654842

[mpr1996-bib-0003] Kirk, N. , Hirsch, E. , Alam, T. , Wakschlag, L. S. , Wiggins, J. L.*, & Roy, A. K.* (2023). A pragmatic, clinically optimized approach to characterizing adolescent irritability: Validation of parent and adolescent reports on the MAPS Temper Loss Scale. International Journal of Methods in Psychiatric Research. e1986. 10.1002/mpr.1986 37702276 PMC10654814

[mpr1996-bib-0004] Wakschlag, L. S. , Sherlock, P. , Blackwell, C. , Burns, J. L. , Krogh‐Jespersen, S. , Gershon, R. C. , Cella, D. , Buss, K. A. , & Luby, J. L. (2023). Modeling the normal:abnormal spectrum of early childhood internalizing behaviors: A clinical‐developmental approach for the multidimensional assessment profiles internalizing dimensions (MAPS‐INT). International Journal of Methods in Psychiatric Research. e1987. 10.1002/mpr.1987 37814600 PMC10654833

[mpr1996-bib-0005] Wiggins, J. L. , Roy, A. K. , & Wakschlag, L. S. (2023). MAPping affective dimensions of behavior: Methodologic advancement of the multidimensional assessment profile scales (MAPS) with pragmatic applications. International Journal of Methods in Psychiatric Research. e1990. 10.1002/mpr.1990 37702271 PMC10654824

[mpr1996-bib-0006] Wiggins, J. L. , Ureña Rosario, A. , MacNeill, L. , Briggs‐Gowan, M. , Smith, J. D. , & Wakschlag, L. S. (2023). Prevalence, stability, and predictive utility of the MAPS clinically optimized irritability score: Pragmatic early assessment of mental disorder risk. International Journal of Methods in Psychiatric Research. e1991. 10.1002/mpr.1991 37728118 PMC10654826

[mpr1996-bib-0007] Wiggins, J. L. , Ureña Rosario, A. , Zhang, Y. , MacNeill, L. , Yu, Q. , Norton, E. , Smith, J. D. , & Wakschlag, L. S. (2023). Advancing earlier transdiagnostic identification of mental health risk: A pragmatic approach at the transition to toddlerhood. International Journal of Methods in Psychiatric Research. e1989. 10.1002/mpr.1989 37723907 PMC10654830

